# The Utilization of Computed Tomography in the Pediatric Emergency Department for Patients With Ventriculoperitoneal Shunts

**DOI:** 10.7759/cureus.56816

**Published:** 2024-03-24

**Authors:** Hamoud Alqarni, Raiyan Almaini, Aiydh Alharbi, Abdullah Aldaffaa, Nawaf Alammari, Omar Alawni, Meshari Dalbouh, Ahmed Alzahrani, Amal Yousif

**Affiliations:** 1 Pediatrics, King Abdulaziz Medical City, Riyadh, SAU; 2 Emergency Medicine, King Abdulaziz Medical City, Riyadh, SAU; 3 Internal Medicine, Prince Sultan Military Medical City, Riyadh, SAU; 4 Pediatric Emergency Medicine, King Abdulaziz Medical City, Riyadh, SAU; 5 Pediatric Emergency Medicine, King Faisal Medical City, Abha, SAU; 6 Medical Imaging, Pediatric Radiology, King Abdulaziz Medical City, Riyadh, SAU; 7 Pediatric Emergency Medicine, King Khalid University Hospital, Riyadh, SAU

**Keywords:** pediatric emergency, shunt malfunction, pediatrics, computed tomography (ct), ventriculoperitoneal (vp) shunt

## Abstract

Introduction

Despite all the advantages of computed tomography (CT) scanning, there is a significant concern due to the rising use of CT scans in children with ventriculoperitoneal (VP) shunts. High doses of radiation are absorbed by patients, raising their chance of acquiring cancer. Evaluating a potential VP shunt malfunction is a frequent encounter in the pediatric emergency room, often necessitating the utilization of a CT scan. This study aims to recognize and quantify the utilization of CT scans in an emergency setting for pediatric patients with a clinical suspicion of VP shunt malfunction.

Methods

This retrospective chart review was conducted on patients who visited the emergency department with suspected VP shunt malfunction in a pediatric tertiary care hospital (King Abdullah Specialist Children Hospital), Riyadh, Saudi Arabia. The study included the files of children between the years 2018 and 2019.

Results

A total of 119 children were included; the main indication for VP shunt insertion was congenital hydrocephalus at 46.8% (n=52). The median number of CT scans done per patient was seven (IQR=3-9). CT findings were abnormal among 55.6% (n=60). The univariate analysis examining the impact of different factors on CT findings showed an association between an abnormal CT finding and female gender (P=0.017), younger age (P=0.03), and the presence of a cerebral cyst (P=0.001); however, subsequent multivariate analysis was not significant for any of these factors. Twenty-two point three percent (n=25) of the patients required neurosurgical intervention, and the associated factors with neurosurgical intervention included changes in activity level (P=0.04), weakness (P=0.004), and altered mental status (P=0.001).

Conclusion

Children with VP shunts are susceptible to significant radiation exposure through the utilization of CT scans whenever they present to the ER with suspected shunt malfunction during their lifespan, which puts them at risk of radiation-related complications, such as cancers. CT imaging remains a helpful tool aiding physicians in making accurate decisions. However, in this study, almost half of the children had unremarkable CT findings. Thus, it is imperative to rationalize its use by establishing tailored guidelines that delineate the appropriate circumstances warranting its application.

## Introduction

Despite the numerous advantages offered by computed tomography (CT) scanning, children with ventriculoperitoneal (VP) shunts are susceptible to substantial radiation exposure through the utilization of CT scans whenever they present to the ER with suspected shunt malfunction during their lifespan, consequently elevating their long-term risk of developing malignancies [1,2]. Additionally, other imaging, such as x-rays (Shunt survey), contribute to the overall risk of radiation-related complications [3,4]. Another alternative to conventional CT scans is low-dose ionizing radiation CT scans, which pose a slightly lower lifetime risk of cancer compared to regular CT scans. Alternatively, the rapid scanning MRI provides a radiation-free option [5]. One CT scan results in a lifetime attributable risk of cancer mortality of 0.07% [6]. Multiple factors contribute to the more noticeable effect of ionizing radiation in children than adults, including higher sensitivities of underdeveloped tissues to ionizing radiation, extended latent period of carcinogenic effect of ionizing radiation, and relatively small body surface area of the pediatric age group [7,8]. In addition, the combination of a large ionizing radiation dose and a higher lifetime risk of ionizing radiation-induced carcinogenesis leads to a much higher lifetime cancer-related mortality in children [9,10].

Evaluation of children with VP shunts is frequently required in emergency settings, likely due to the risk of shunt failure, which often outweighs the risk of radiation exposure. Cohen et al. found that a significant proportion of CT scans ordered in the ER to rule out suspected shunt malfunction demonstrated no association between the observed clinical symptoms and signs and the occurrence of radiological shunt malfunction [11]. Studies have shown that, in the first 10 years following the insertion of VP shunts in the pediatric age group, an average of 8.5 CT scans are performed per patient. Almost 50% of the ER visits suspecting shunt malfunction during the study have required a CT image of the head; however, only 17%-20% end up with significant findings requiring additional surgery [12,13]. During evaluating shunt malfunction in the ER, children with VP shunt undergo a shunt series X-ray of the skull, cervical spine, chest, and abdomen alongside head CT, which increases their accumulative risk of radiation-related complications. Another method to decrease radiation exposure is to reduce the number of slices in use [14,15]. Sainte-Rose et al. reduced CT scan slices to only four slices (from 64-slice and 16-slice CT scanners). The targets for the selected slices were the lateral ventricles, basal ganglia, and third and fourth ventricles. There were no cases of missing increases in ventricular size by using a limited CT scan, which matched the final report (looking at all the slices) by 95.7% [16]. Moreover, Alhilali et al. concluded that reducing the number of slices would have avoided 90% of the radiation dose while detecting most, if not all, shunt failure-related issues [17]. Ultrasound can alternatively be used instead of CT, as demonstrated by Albert et al., who found that infants acquired 2.76 mSv while being followed up for shunts, which could have been saved if they had been followed up with cranial ultrasound [18]. Other options include rapid MRI and optic nerve sheath diameters (ONSD) measurements, which are generally unavailable and user-dependent in an emergency department [18].

Therefore, steps to reduce the risk of long-term ionizing radiation consequences are crucial in children with a VP shunt, given their vulnerability to undergo multiple CT scans later in life. This research aims to recognize and quantify the utilization of CT scans in an emergency setting for pediatric patients with clinical suspicion of VP shunt malfunction. We recommend introducing a national CT shunt protocol for all radiology units. A combined protocol reducing slice number and tube current could significantly reduce radiation exposure. However, to facilitate decisions, it would be most effective to decrease the use of CT, as it should always be shunt protocol and should not be used if the threshold of suspicion is low and the child could be admitted for observation instead. Sufficient education of patients and next-of-kin is also essential to avoid radiophobia and ensure that the proper investigation is used for the correct indication, which can sometimes mean choosing a CT scan where applicable.

## Materials and methods

Subjects and methods

This is a retrospective chart review of patients' medical records who visited the ER with suspected VP shunt malfunction in a tertiary specialist children's hospital (King Abdullah Specialist Children Hospital), Riyadh, Saudi Arabia. All patient files that underwent a CT scan for the suspicion of VP shunt malfunction between the years 2018 and 2019 were included. The study included all patients aged 0-14 years. Patients’ charts were reviewed by the research team. Data collected include patients’ demographic data (e.g., age and gender). Additionally, clinical symptoms and signs associated with VP shunt malfunction were collected from the emergency visit report, which includes a change in the level of activity, abdominal pain, ataxia, seizure, weakness, fever, vomiting, headache, altered mental status, bulging fontanelle, dizziness, and change in behavior. Results of medical imaging done during the visit were reviewed, and patients were considered to have abnormal CT findings if there was an interval increase in ventricle size (i.e., ventriculomegaly) or if there was an abnormality in the VP shunt system, such as obstruction. Shunt surveys done during the visit were acquired and were considered abnormal if it had a fracture, kinking, obstruction, or leaking of the tube. Data of patients who required further imaging by MRI were recorded. Then, we recorded if the patient with abnormal CT findings underwent corrective neurosurgical intervention within 30 days, which included revision, removal, replacement, or externalization. 

Statistical analysis

Statistical analysis was performed using RStudio (version 4.3.0; RStudio Team, Boston, MA). Categorical data, such as gender, were presented as frequencies and percentages, whereas numerical data, such as age, were expressed as median and interquartile ranges (IQRs). The association between requiring neurosurgical intervention and patients’ characteristics was assessed using Pearson’s chi-squared test, Fisher’s exact test for categorical data, or a Wilcoxon rank sum test for continuous data. The same tests were applied to assess factors associated with having abnormal CT findings. Results of the inferential analysis were further used to construct a multivariable logistic regression model to determine the predictors of requiring a neurosurgical intervention or having abnormal CT findings (each in a separate model). Results were presented as odds ratios (ORs) and 95% confidence intervals (CIs). A p-value of < 0.05 indicated statistical significance.

## Results

Demographic and clinical characteristics

The current study analyzed data from 119 pediatric patients who underwent CT scans for a suspected VP shunt malfunction. A total of 80 patients were males (67.2%). The median age of patients when CT was obtained was 12.0 months (IQR = 5.0-48.0), and the median age upon shunt placement was 3.0 months (IQR = 2.0-8.0). The most common reasons for shunt placement were congenital hydrocephalus in 52 patients (46.8%) and neoplasms in 14 patients (12.6%). A total of 18 children had changes in their activity levels (24.3%). The most frequently reported symptoms/signs upon presentation were vomiting in 54 patients (65.1%) and headache in 43 patients (44.3%). More details about the demographic and clinical characteristics are listed in Table 1.

**Table 1:** Demographic and clinical characteristics of patients (N=119). *n (%) for categorical variables; median (IQR) for continuous variables **Others include leaking or filling defect Abbreviations: CT: Computed Tomography, VP: Ventriculoperitoneal, ER: Emergency Room

Characteristic	Missing	Description*
Gender	0 (0%)	
Male		80 (67.2%)
Female		39 (32.8%)
Age when the CT was obtained	5 (4.2%)	12.0 (5.0-48.0)
Reason for VP shunt insertion	8 (6.7%)	
Congenital hydrocephalus		52 (46.8%)
Obstructive hydrocephalus		7 (6.3%)
Myelomeningocele		9 (8.1%)
Trauma		6 (5.4%)
Neoplasms		14 (12.6%)
Meningitis		7 (6.3%)
Cerebral cyst		8 (7.2%)
Others**		8 (7.2%)
Age when the VP shunt was placed	65 (55%)	3.0 (2.0-8.0)
Number of ER visits due to VP shunt malfunction	7 (5.9%)	3.0 (2.0-5.0)
Number of ER visits for suspected VP shunt malfunction that required doing CT	7 (5.9%)	3.0 (2.0-5.0)
Change in level of activity	45 (38%)	18 (24.3%)
Symptoms and signs associated with VP shunt malfunction		
Abdominal pain	45 (38%)	9 (12.2%)
Ataxia	47 (39%)	3 (4.2%)
Seizures	32 (27%)	34 (39.1%)
Weakness	49 (41%)	16 (22.9%)
Fever	44 (37%)	22 (29.3%)
Vomiting	36 (30%)	54 (65.1%)
Headache	22 (18%)	43 (44.3%)
Altered mental status	48 (40%)	6 (8.5%)
Bulging fontanelle	48 (40%)	6 (8.5%)
Dizziness	45 (38%)	11 (14.9%)
Change in behavior	46 (39%)	4 (5.5%)

Radiological characteristics

Out of patients under study, CT findings were available for 108 patients. Of them, abnormal findings were revealed among 60 patients (55.6%). The median number of CT scans performed was 7.0 per patient within the patient's lifetime during the study period (IQR = 3.0-9.0). Plain radiographs (shunt survey) were done for 94 patients (83.9%), and unremarkable findings were reported in 82 patients (88.2%). MRI scans were performed for 37 children (33.0%), all of whom underwent MRI brain, and five children (18.5%) underwent MRI spine (Table 2).

**Table 2:** Radiological characteristics of patients under study (N=119). *n (%) for categorical variables; median (IQR) for continuous variables **Abnormal findings related to VP shunt malfunction include worsening ventriculomegaly or the apparent abnormal VP shunt system §Descriptive analyses are based on 94 records of patients who had a shunt survey done for the same presentation ¥ Descriptive analyses are based on 37 records of patients who had an MRI done for the same presentation Abbreviations: CT: Computed Tomography, MRI: Magnetic Resonance Imaging

Characteristic	Missing	Description*
CT finding	11 (9.2%)	
Normal		34 (31.5%)
Abnormal**		60 (55.6%)
Other		14 (13.0%)
Number of CT scans done for VP shunt malfunction	8 (6.7%)	7.0 (3.0-9.0)
Patient age at CT (years)	0 (0%)	1.0 (0.30-4.0)
Shunt survey done for the same presentation	7 (5.9%)	94 (83.9%)
Shunt survey findings^§^	1 (1.1%)	
Fractured tube		0 (0.0%)
Kinking of the tube		4 (4.3%)
Obstruction of the tube		3 (3.2%)
Unremarkable		82 (88.2%)
Others (leak & decrease filling)		4 (4.3%)
MRI done	7 (5.9%)	37 (33.0%)
MRI types^¥^	10 (27%)	
MRI brain		27 (100%)
MRI spine		5 (18.5%)
MRI with contrast		4 (14.8%)

Characteristics of neurosurgical interventions

**Figure 1:**
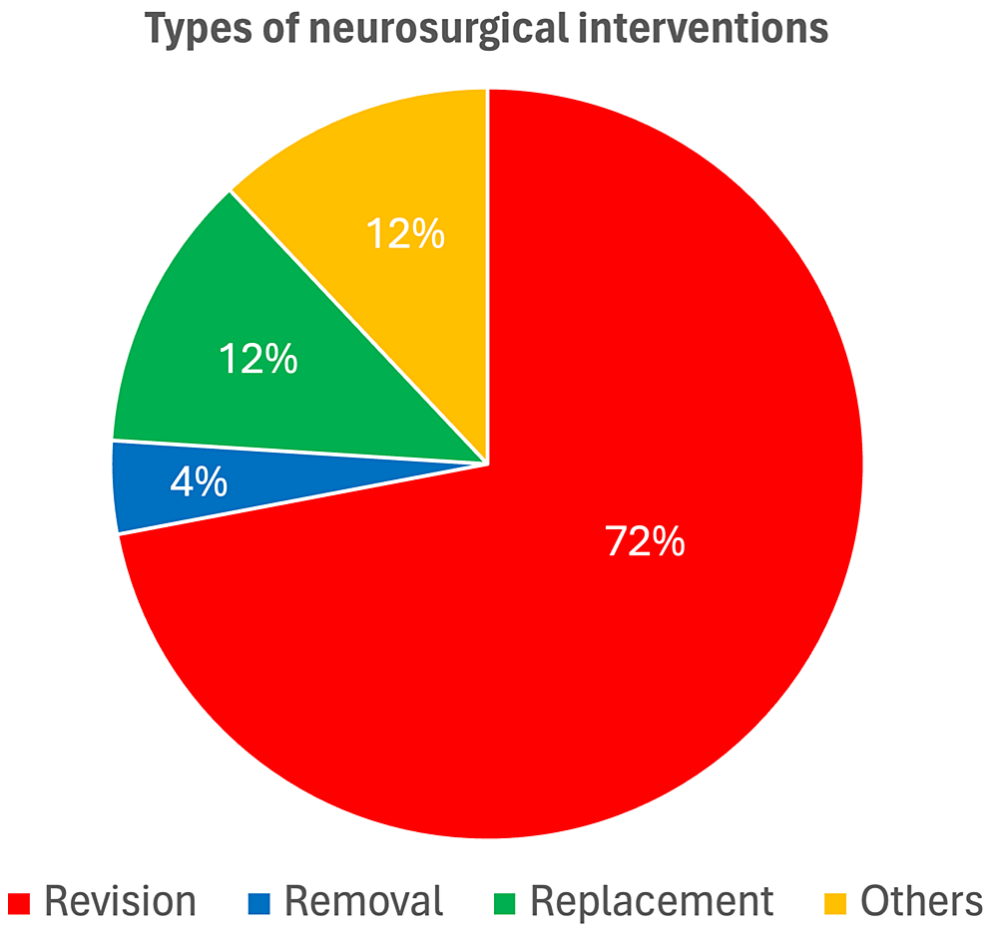
The proportions of types of corrective neurosurgical interventions within 30 days (N=25). Others include decompression and cyst fenestration.

In general, data regarding the need for a neurosurgical intervention were available for 112 patients, of whom 25 patients required an intervention (22.3%). Surgeries were performed after a median of 1.0 days (IQR = 1.0-5.0) following CT scans. Revisions were performed for 18 patients (72.0%), whereas replacement and removal surgeries were performed for three patients (12.0%) and one patient (4.0%), respectively. Additionally, three patients (12.0%) underwent other surgical interventions, including decompression and cyst fenestration (Figure 1).

Factors associated with requiring a neurosurgical intervention

The need for a neurosurgical intervention was significantly higher among those who had changes in activity levels (seven out of 18 (38.9%) vs 8 out of 56 (14.3%), p = 0.040), weakness (eight out of 16 (50.0%) vs seven out of 54 (13.0%), p = 0.004), fever (eight out of 22 (36.4%) vs seven out of 55 (13.2%), p = 0.030), headache (17 out of 43 (39.5%) vs six out of 54 (11.1%), p = 0.001), altered mental status (five out of 6 (83.3%) vs 10 out of 65 (15.4%), p = 0.001), and dizziness (eight out of 11 (72.7%) vs nine out of 63 (14.3%), p < 0.001). Additionally, significantly higher proportions of children with abnormal CT findings required a neurosurgical intervention (18 out of 60 (30.0%) vs one out of 34 (2.9%), p = 0.001) (Table 3).

**Table 3:** Factors associated with requiring a neurosurgical intervention (N=112). ^1^n (%); median (IQR) ^2^Pearson’s chi-squared test; Wilcoxon rank sum test; Fisher’s exact test Abbreviations: CT: Computed Tomography, VP: Ventriculoperitoneal, ER: Emergency Room

Characteristic	Missing	Required a neurosurgical intervention		p-value^2^
		No, N=87^1^	Yes, N=25^1^	
Gender	0 (0%)			0.537
Male		58 (79.5%)	15 (20.5%)	
Female		29 (74.4%)	10 (25.6%)	
Age when the CT was obtained	0 (0%)	12.0 (5.0-44.0)	48.0 (4.0-72.0)	0.206
Reason for VP shunt insertion	2 (1.8%)			0.459
Congenital hydrocephalus		40 (78.4%)	11 (21.6%)	
Obstructive hydrocephalus		4 (57.1%)	3 (42.9%)	
Myelomeningocele		7 (77.8%)	2 (22.2%)	
Trauma		6 (100.0%)	0 (0.0%)	
Neoplasms		9 (64.3%)	5 (35.7%)	
Meningitis		7 (100.0%)	0 (0.0%)	
Cerebral cyst		6 (75.0%)	2 (25.0%)	
Others		6 (75.0%)	2 (25.0%)	
Age when the VP shunt was placed	58 (52%)	5.0 (2.0-9.0)	3.0 (1.0-3.0)	0.358
Number of ER visits due to VP shunt malfunction	1 (0.9%)	3.0 (2.0-5.0)	3.0 (1.0-8.0)	0.443
Number of ER visits for suspected VP shunt malfunction that required doing CT	0 (0%)	3.0 (2.0-4.0)	3.0 (1.0-9.0)	0.410
Change in level of activity	38 (34%)			0.040
No		48 (85.7%)	8 (14.3%)	
Yes		11 (61.1%)	7 (38.9%)	
Abdominal pain	38 (34%)			0.076
No		54 (83.1%)	11 (16.9%)	
Yes		5 (55.6%)	4 (44.4%)	
Ataxia	40 (36%)			0.509
No		55 (79.7%)	14 (20.3%)	
Yes		2 (66.7%)	1 (33.3%)	
Seizures	25 (22%)			0.843
No		43 (81.1%)	10 (18.9%)	
Yes		27 (79.4%)	7 (20.6%)	
Weakness	42 (38%)			0.004
No		47 (87.0%)	7 (13.0%)	
Yes		8 (50.0%)	8 (50.0%)	
Fever	37 (33%)			0.030
No		46 (86.8%)	7 (13.2%)	
Yes		14 (63.6%)	8 (36.4%)	
Vomiting	29 (26%)			0.131
No		26 (89.7%)	3 (10.3%)	
Yes		41 (75.9%)	13 (24.1%)	
Headache	15 (13%)			0.001
No		48 (88.9%)	6 (11.1%)	
Yes		26 (60.5%)	17 (39.5%)	
Altered mental status	41 (37%)			0.001
No		55 (84.6%)	10 (15.4%)	
Yes		1 (16.7%)	5 (83.3%)	
Bulging fontanelle	41 (37%)			>0.999
No		53 (81.5%)	12 (18.5%)	
Yes		5 (83.3%)	1 (16.7%)	
Dizziness	38 (34%)			<0.001
No		54 (85.7%)	9 (14.3%)	
Yes		3 (27.3%)	8 (72.7%)	
Change in behavior	39 (35%)			>0.999
No		55 (79.7%)	14 (20.3%)	
Yes		3 (75.0%)	1 (25.0%)	
CT finding	4 (3.6%)			0.001
Normal		33 (97.1%)	1 (2.9%)	
Abnormal		42 (70.0%)	18 (30.0%)	
Other		9 (64.3%)	5 (35.7%)	
Was the shunt survey done for the same presentation?	0 (0%)			>0.999
No		14 (77.8%)	4 (22.2%)	
Yes		73 (77.7%)	21 (22.3%)	

In the regression analysis, we incorporated the significantly associated variables from the inferential analysis as independent variables in the multivariable logistic regression model. Requiring a neurosurgical intervention was used as a dependent variable. However, we excluded three independent variables from the model due to the high risk of multicollinearity (a variance inflation factor > 5). These variables included changes in activity levels, dizziness, and CT findings. The regression model results showed no significant predictors of undergoing a neurosurgical intervention.

Factors associated with having abnormal CT findings

We analyzed the factors associated with CT findings among 108 patients with available CT results. The univariate analysis showed that females had significantly higher proportions of abnormal CT findings (27 (45.0%) vs 11 (22.9%), p = 0.017). Additionally, children with abnormal CT were significantly younger (median age = 9.0 years, IQR = 3.0-48.0 vs median = 14.0 years, IQR = 5.0-48.0, p = 0.037). Notably, the proportions of children with abnormal CT findings were significantly higher among those with congenital hydrocephalus (35 (60.3%) vs 15 (29.2%), p = 0.001) and were significantly lower among those with a cerebral cyst (0 (0.0%) vs 8 (16.7%), p = 0.001) (Table 4). However, on the multivariable analysis, no significant risk factors were revealed for having abnormal CT findings.

**Table 4:** Factors associated with having abnormal CT findings. Abbreviations: CT: Computed Tomography, VP: Ventriculoperitoneal, ER: Emergency Room, MRI: Magnetic Resonance Imaging

Characteristic	Missing	MRI findings		p-value
		Normal N=48	Abnormal N=60	
Gender	0 (0%)			0.017
Male		37 (77.1%)	33 (55.0%)	
Female		11 (22.9%)	27 (45.0%)	
Age when the CT was obtained	0 (0%)	14.0 (5.0-48.0)	9.0 (3.0-48.0)	0.037
Reason for VP shunt insertion				
Congenital hydrocephalus	2 (1.9%)	14 (29.2%)	35 (60.3%)	0.001
Obstructive hydrocephalus	2 (1.9%)	3 (6.3%)	4 (6.9%)	>0.999
Myelomeningocele	2 (1.9%)	5 (10.4%)	4 (6.9%)	0.728
Trauma	2 (1.9%)	3 (6.3%)	3 (5.2%)	>0.999
Neoplasms	2 (1.9%)	9 (18.8%)	4 (6.9%)	0.064
Meningitis	2 (1.9%)	2 (4.2%)	4 (6.9%)	0.687
Cerebral cyst	2 (1.9%)	8 (16.7%)	0 (0.0%)	0.001
Age when the VP shunt was placed	56 (52%)	5.0 (2.0-11.0)	3.0 (1.0-8.0)	0.455
Number of ER visits due to VP shunt malfunction	1 (0.9%)	3.0 (2.0-7.5)	3.0 (1.0-5.0)	0.130
Number of ER visits for suspected VP shunt malfunction that required doing CT	0 (0%)	3.0 (2.0-7.0)	3.0 (1.0-4.3)	0.406
Change in level of activity	36 (33%)	4 (14.3%)	14 (31.8%)	0.094
Abdominal pain	36 (33%)	2 (6.9%)	7 (16.3%)	0.297
Ataxia	38 (35%)	1 (3.6%)	2 (4.8%)	>0.999
Seizures	23 (21%)	12 (34.3%)	21 (42.0%)	0.473
Weakness	40 (37%)	6 (23.1%)	10 (23.8%)	0.945
Fever	37 (34%)	6 (20.7%)	14 (33.3%)	0.244
Vomiting	28 (26%)	21 (63.6%)	32 (68.1%)	0.679
Headache	15 (14%)	20 (48.8%)	20 (38.5%)	0.318
Altered mental status	38 (35%)	0 (0.0%)	6 (14.0%)	0.075
Bulging Fontanelle	39 (36%)	2 (7.4%)	4 (9.5%)	>0.999
Dizziness	36 (33%)	5 (17.2%)	6 (14.0%)	0.747
Change in behavior	37 (34%)	1 (3.7%)	3 (6.8%)	>0.999
How many CT scans has the patient done for the indication of VP shunt malfunction during the time period of the study?	1 (0.9%)	7.0 (3.0-9.3)	7.0 (3.0-9.0)	0.680
Was the shunt survey done for the same presentation?	0 (0%)	41 (85.4%)	49 (81.7%)	0.603
Was an MRI done for this patient?	2 (1.9%)	16 (34.0%)	20 (33.9%)	0.988

## Discussion

The use of CT in the emergency department has increased; it is a common indication in the pediatric emergency department to assess possible complications related to VP shunt [11]. Suspected shunt malfunction is a frequent indication for emergency neuroimaging. The literature has few previous studies that reported the utilization of CT scans in the emergency department for children who presented with the suspicion of VP shunt malfunction, especially in Saudi Arabia. Therefore, in this study, we assessed the number of CTs performed among children with VP shunt and factors associated with its findings. The number of emergency visits for VP shunt malfunction that required CT ranged between two and five visits, with a mean of three visits. According to Florin et al., in a review of 1319 children with VP shunt, they found that those children visited the ER 6,636 times over a 10-year follow-up and reported that 49.4% of all emergency department visits underwent CT [13]. A study conducted on 152 children with VP shunt reported that the range of CT scans for each child was 0-20 scans with a mean of 3.33 CT scans [14]. However, it should be noted that each study estimated the number of CT scans differently. It was reported that children with a shunt are exposed to a large number of imaging (CT) that deliver radiation [12]. Congenital hydrocephalus was the primary cause of performing VP shunt insertion among the children, which is similar to the study done by Cohen et al. [11].

The rapid diagnosis of VP shunt malfunction is required to prevent morbidity and mortality. The conventional symptoms of VP shunt malfunction include vomiting, headache, irritability, drowsiness, and somnolence [15]. In this study, the children who experienced VP shunt malfunction majorly complained of vomiting, headache, and seizures. Such symptoms were more reported among those who experienced VP shunt malfunction. On the other hand, change in behavior and ataxia were the least reported symptoms. Cohen et al. revealed that none of the symptoms and signs of VP malfunction, including vomiting, dizziness, altered mental status, headache, and seizure, are significantly associated with VP shunt malfunction [11]. In this study, symptoms and signs were reported, with univariate analysis showing an association between VP shunt malfunction and being female, younger age, and underlying cerebral cyst; however, in the multivariable analysis, no significant association was revealed for having abnormal CT findings and any of the symptoms and signs, which also agrees with Cohen et al. [11].

The symptoms of VP shunt malfunction can mimic other pediatric conditions; hence, emergency physicians rely potentially on cranial imaging to discriminate patients with VP shunt malfunction. On the other hand, other physicians use MRI for malfunction diagnosis [19]. In the present study, more than 50% of children who underwent CT displayed abnormal findings, which is an interval increase in ventricle size. The number of CTs performed for VP shunt malfunction ranged between three to nine, with a mean of seven, and only 37 patients underwent MRI.

Almost 25-40% of shunts fail in the first year following insertion, and 56-80% of patients experience malfunctions 10 years after shunt placement [20-23]. Managing shunt malfunction often requires revision of the shunt, estimated in the US as 16,000 shunt revisions annually [23]. In our study, less than a quarter of the patients required neurosurgical intervention, and most of those who performed the intervention required revision. The univariate analysis revealed that the factors associated with neurosurgical intervention included changes in activity level, weakness, fever, altered mental status, dizziness, and abnormal CT findings. However, multivariate analysis revealed that weakness, fever, headache, or altered mental status were not risk factors for neurological intervention. In a previous study, it was reported that neurosurgical intervention within 30 days was positively associated with behavioral change and bulging fontanelle, whereas the intervention was negatively associated with fever and seizure [11]. According to Florin et al., out of 3,275 ER visits due to VP shunt malfunction, which needed CT, 653 patients (19.9%) were associated with a shunt revision [13].

This study has limitations. As patients' data were collected by reviewing medical records, some of the variables, especially symptoms and signs, were not documented. Missing data and a small sample size in a single hospital contributed to difficult inferential multivariant analysis. Nevertheless, we believe that shedding light on this issue will help fellow physicians and researchers identify the gaps and suggest improvements for future studies, which will help in establishing guidelines that rationalize the use of CT scans during the assessment of VP shunt malfunction in an emergency setting by providing specific or relative indications, thus ensuring better patient care.

## Conclusions

Pediatric patients with VP shunt undergo several CT scans, which put them at an increased risk of cancer due to radiation exposure. The major symptoms that were common among patients with VP shunt malfunction included vomiting, headache, and seizure. The findings of CT were abnormal among almost half of the patients, and we could not find any factor associated with CT abnormal findings. Additionally, we could not find any risk factor for requiring neurosurgical interventions.

We advise a national VP shunt imaging guideline/protocol that clarifies the need for emergency imaging and recommends the implementation of low radiation exposure imaging protocols that protect these children, particularly those prone to multiple imaging because of their VP shunts. Reduced CT slices with low radiation doses are an option; however, rapid MRI scanning is optimum for these children, and we hope they are available for such cases.
